# Sterility, Result of Lithotomy

**Published:** 1874-10

**Authors:** 


Sterility as a Result of Lithotomy.—At a meeting of the Medical Society of London, Mr. Teevan related four cases:

1. A hall keeper, aged, 44, was cut by the lateral operation, at a provincial hospital, twenty years ago. He married three years afterwards; but his wife, although his junior, had never had a child or a miscarriage. The patient stated that he had no emissions during connection.

2. A painter, aged 47, married when thirty-one years old, Lateral lithotomy was performed on him three years afterwards. His wife bore him two children before, but none subsequently to the operation. He had quite lost the faculty of emission during coitus.

3. A shoemaker, aged 45, was cut by the lateral method when two years old. He married when 25; but his wife, although his junior, had had no child or miscarriage. The patient had no emissions during connection.

4. A shipwright, aged 45, had lateral lithotomy performed on him when four years old. He was married at the age of 23, his wife being his junior. She had borne him no family. He had no emission during coitus. The cause of sterility was very clear; for lithotomy, as usually performed, involved a laceration of the floor of the prostatic portion of the urethra, and obliteration of the orifices of the ejaculatory ducts. Sometimes the prostate split the roof, and the infirmity was obviated.—*Medical and Surgical Reporter—August.*

